# Exploitation of cholesterol-dependent cytolysins for targeted biosensing and therapeutic systems

**DOI:** 10.1007/s13346-026-02128-3

**Published:** 2026-05-04

**Authors:** Henry O. Abanum, Ethan Watt, Emmanuel A. Ho

**Affiliations:** 1 School of Pharmacy, University of Waterloo, 10 Victoria St. S, Kitchener, ON N2G 1C5 Canada; 2 Waterloo Institute for Nanotechnology, Waterloo, Canada

**Keywords:** Cholesterol-dependent cytolysins, Pore-forming toxins, Targeted drug delivery, Biosensing, CDC-responsive nanocarriers, Translational medicine

## Abstract

Cholesterol-dependent cytolysins (CDCs) represent a unique and structurally conserved family of pore-forming toxins secreted by Gram-positive bacterial pathogens. These exotoxins significantly contribute to the virulence and pathogenicity of many CDC-expressing organisms. CDCs selectively recognize and bind cholesterol-rich membranes in host cells and undergo subsequent conformational changes leading to pore formation. Beyond their role as virulence factors, their unique membrane-targeting and pore-forming mechanisms have enabled promising avenues in precision biosensing and targeted therapeutic delivery. Recent advances in nanotechnology and biotechnology have enabled diverse and pivotal biomedical applications of CDCs, which encompass their exploitation as potential bacterial vaccine candidates, ligands for targeted drug delivery, biosensors for pathogen detection, and CDC-responsive nanocarriers for targeted therapeutic delivery. This review explores these emerging roles while highlighting critical factors for the design and evaluation of CDC-responsive platforms, as well as strategies for characterizing their interactions with biological systems. While several preclinical models have demonstrated promising in vitro and in vivo data, the clinical translation of CDC-associated therapies remains hindered by challenges which affect their widespread biomedical application. Understanding the biological mechanisms and molecular interactions of CDCs, along with the effective optimization of the physicochemical properties of CDC-targeted nanomaterials, holds great prospect in overcoming these challenges. Future research focusing on enhanced understanding of CDC physiological interactions and developing innovative CDC-responsive nanoplatforms for targeted delivery offers valuable prospects in advancing this field and may ultimately enable the successful clinical translation of CDC-based therapies. Despite current challenges in ongoing research, the strategic exploitation of CDCs holds significant potential in the advancement of targeted biosensing and therapeutic innovations.

## Introduction

**Fig. 1:**
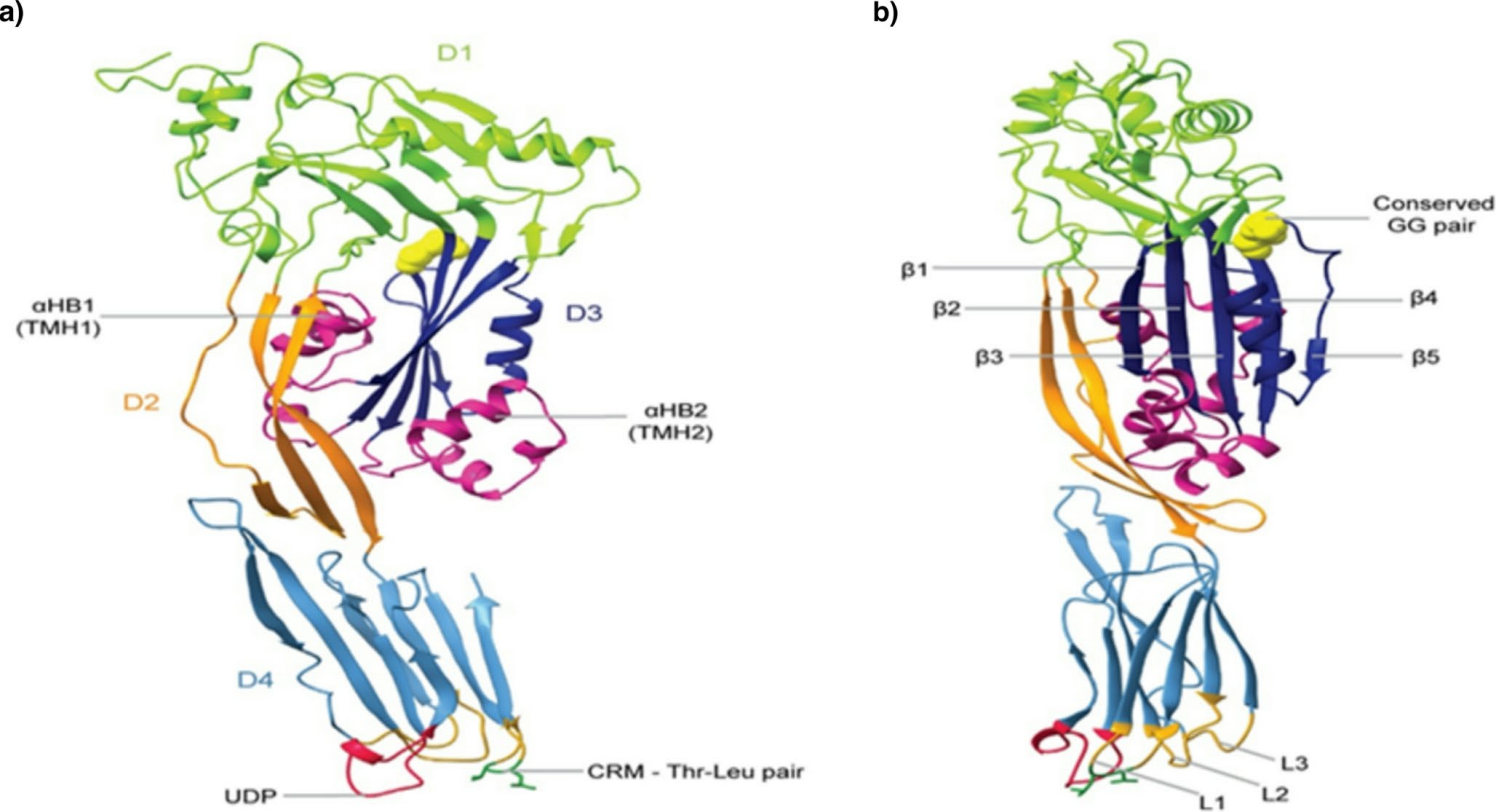
The structure of a CDC monomer. (**a**) Perfringolysin O (PFO) monomeric structure (based on Protein Data Bank (PDB) ID: 1PFO) showing its four domains - D1, D2, D3, and D4 - colored green, orange, navy, and blue, respectively. Key functional features are also highlighted: the transmembrane hairpin (TMH) regions (αHB1 and αHB2) appear in pink, the UDP is shown in red, and the CRM is marked in dark green. (**b**) A side view of the PFO structure, rotated 90° along the y-axis, reveals the core β-strands in domain D3 (β1 to β5). The conserved glycine pair in D3 is highlighted in yellow using a Corey-Pauling-Koltun color scheme (CPK) coloring, and the L1 to L3 loops are shown in gold. *Reproduced from Johnstone et al.* [4], ©*2022 The Authors. Licensed under CC BY-NC. Published by Wiley Periodicals LLC on behalf of IUBMB*

The largest class of bacterial toxins are known as pore-forming toxins. They are comprised of numerous protein exotoxins produced by a variety of pathogenic bacteria. As the name suggests, they act by destroying the cellular barrier through the formation of pores to enable microbial colonization and dissemination [1]. A major subset of this class is the family of CDCs, which are secreted pore-forming toxins that specifically target membrane cholesterol to aid in overall pathogenesis of the microbe. The structural conformation and pore kinetics of these protein toxins have been well documented previously and are largely conserved among all members of the family [2]. CDCs assemble into oligomeric complex monomers, which are comprised of four domains (Fig. 1). The first domain (D1) and third domain (D3) are often considered together as a membrane attack complex/perforin (MACPF) domain, while the second domain (D2) serves as a flexible hinge to link this complex to the fourth domain (D4). D4 contains the undecapeptide (UDP) membrane-sensing loop, the cholesterol-recognition motif (CRM), and three additional loops (L1 to L3), that collectively serve to mediate CDC binding to host membranes [3].

**Fig. 2:**
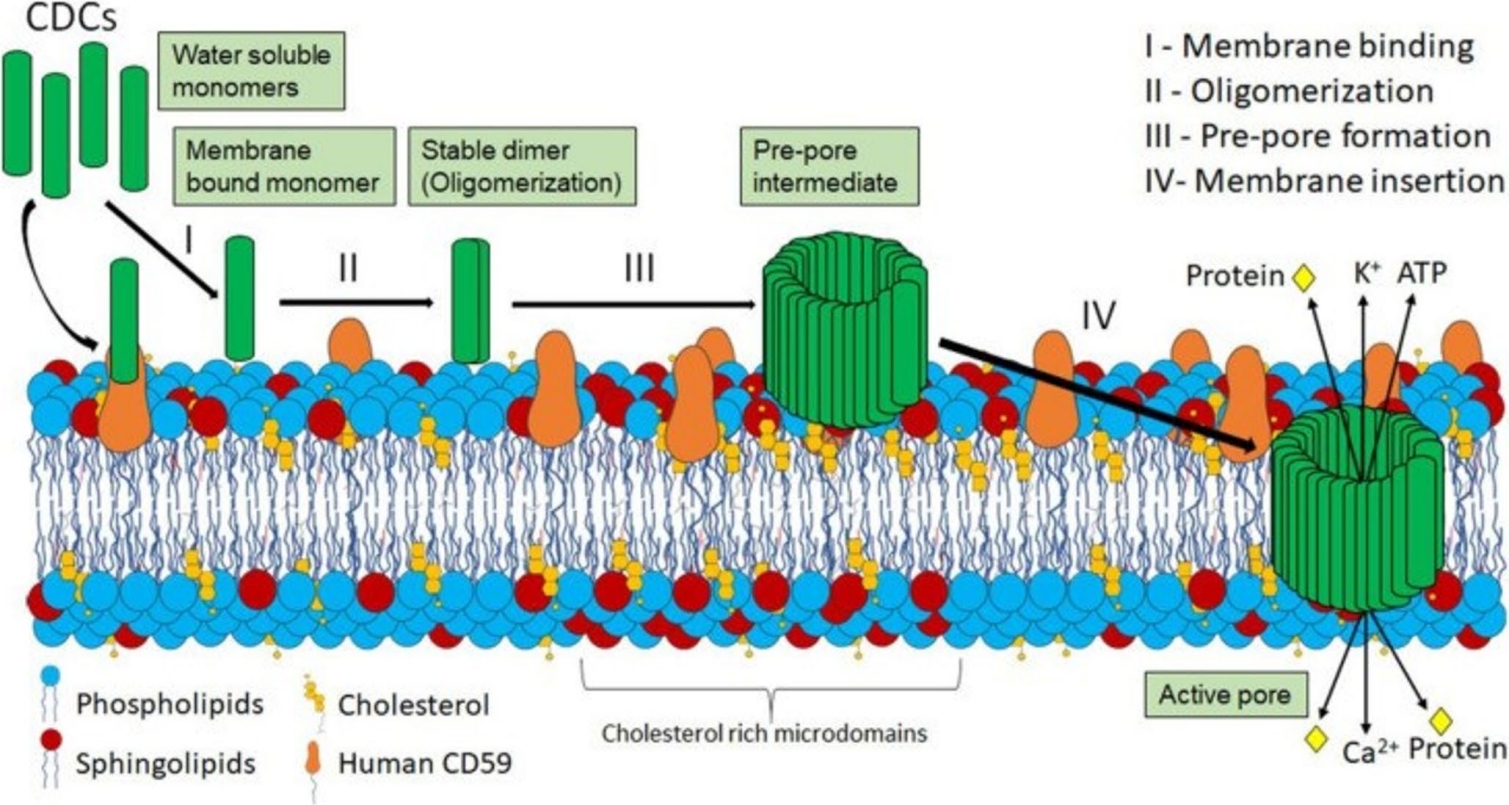
Mechanism of CDC pore-formation. Reproduced from Thapa et al. [6], licensed under CC BY 4.0

Upon perpendicular membrane binding facilitated by this motif, changes in the monomer structure lead to subsequent intermolecular contacts with the attached membrane to form a ring-like oligomeric prepore consisting of 34–50 subunits [5]. Following oligomerization and prepore formation (Fig. 2), a vertical collapse of the complex brings the monomer closer to the target membrane, at which point α-helical bundles unfurl to produce two adjacent β-strands to form a large β-barrel transmembrane pore with a large internal diameter of about 250 Å [4].

These toxins are produced by a wide array of species from the *Arcanobacterium*, *Bacillus*, *Brevibacillus*, *Clostridium*, *Gardnerella*, *Lactobacillus*, *Listeria*, *Paenibacillus*, and *Streptococcus* genera [7]. Typical functions of CDCs include membrane disruption, which facilitate bacterial growth and dissemination. While CDC intoxication can lead to immune invasion, it also commonly results in cell lysis, and can induce programmed cell death mechanisms such as apoptosis, pyroptosis, and oncosis, as well as activate various signaling pathways that further support bacterial spread [6]. Immune evasion can be achieved by the toxins through direct killing of immune cells, but can also lead to their suppression through impairment of macrophage responses, reduction of phagocytosis, or limiting activation and oxidative burst of neutrophils [8]. As a result, these toxins are key virulence factors that are in many cases necessary for the development of severe bacterial infections such as meningitis, pneumoniae, and necrotic tissue infections [9].

Recent advances in nanotechnology have enabled the strategic use of biological molecules, including CDCs, as functional components in drug delivery systems. Owing to the unique properties of CDCs, their conserved cholesterol recognition, and pivotal role in infection for a variety of pathogens, they have naturally emerged as powerful therapeutic molecules. Their pore-forming activity, cell-type specificity, and structural predictability have made them a promising class of molecules for a range of biomedical and therapeutic applications. By exploiting their cholesterol-dependent binding and associated structural transitions, CDCs can be incorporated into nanoparticle platforms to enable triggered drug release, targeted membrane permeabilization, and biosensing applications. Recent studies have explored the application of CDCs in drug delivery, biosensing, and targeted cytolysis for therapeutic benefits, leveraging their native mechanisms and role in pathogenesis. These studies underscore the emerging potential of CDC-based nanoplatforms for selective delivery of therapeutic agents and localized cytolytic activity in cancer and infectious disease models [10, 11]. These findings highlight the growing potential of CDCs as components of stimuli-responsive and targeted delivery systems. As such, this review aims to identify the current applications of CDCs in literature and highlight key factors and challenges towards the development and characterization of future toxin-responsive systems.

## Biomedical applications of CDCs

CDCs represent a family of pore-forming toxins produced by various Gram-positive bacteria [12]. Beyond their primary role in bacterial virulence and pathogenesis, they have been exploited for diverse biomedical applications due to their unique ability to interact with cholesterol-rich membranes. For example, CDCs have been investigated as potent bacterial vaccine candidates due to their ability to elicit protective immunogenic responses [13]. CDCs such as pneumolysin (PLY) from *Streptococcus pneumoniae*, and listeriolysin O (LLO) from *Listeria monocytogenes* have been investigated as components in vaccine development. In the past years, incorporation of detoxified forms of PLY into pneumococcal vaccines have been reported to enhance efficacy by inducing antibodies that promote complement deposition on pneumococcal strains [14]. Similarly, LLO has been utilized in attenuated strains of *Listeria monocytogenes* for vaccine delivery platforms, utilizing the ability of LLO to facilitate antigen presentation by disrupting phagosomal membranes and enhance the presentation of antigens [15]. The immunogenic properties of CDCs, together with their selective membrane disrupting capability, provide an opportunity for their use in stimuli-responsive vaccine delivery platforms. In such systems, CDCs can facilitate controlled antigen release and improve intracellular delivery, thereby enhancing vaccine efficacy and targeted immune responses.

CDCs can also serve as potential ligands for therapeutic applications. Their high binding affinity for cholesterol-rich membranes have been exploited for targeted drug delivery strategies. Coupling or conjugating CDCs with therapeutic agents can facilitate endosomal escape, with the aims to improve the specificity and efficacy of treatments while minimizing off-target effects [16]. Beyond this, they have shown potential as biomarkers for the detection of specific bacterial infections. The presence of CDCs or specific antibodies that inhibit them in biological samples, has been used to detect the presence and growth of CDC-expressing bacterial infections through specific diagnostic assays [17].

As a result of their unique role in bacterial pathogenesis, CDCs have also become useful targets for therapeutic inhibition. These entails the deliberate blocking or suppression of CDCs to prevent or treat CDC-expressing bacterial infections without disrupting normal physiological functions. A typical example is the use of small molecule inhibitors to prevent CDC binding or pore formation [18]. The use of cholesterol-rich nanoparticles to sequester CDCs have also displayed promise as a therapeutic approach via toxin neutralization strategies [19]. These techniques help to prevent tissue necrosis during active infections and enhance antimicrobial effectiveness and efficacy.

The ability of CDCs to drive stable transmembrane pores into cholesterol-rich host cell membrane have been exploited for controlled release targeted drug delivery. The presence of viable CDCs in delivery systems are capable of triggering the release of encapsulated drug in response to the presence of cholesterol-rich membranes [20, 21]. This approach offers an emerging strategy for the design of responsive drug delivery platforms to potentially improve treatment outcomes. CDCs interactions with cholesterol-rich membrane and pore forming capability have made them promising targets for potential exploitation. Ongoing research in the field of microbiology and bacterial pathogenesis, immunology, vaccine development and therapeutics, nanomedicine and drug delivery, continues to uncover promising innovative ways to exploit these unique toxins for both therapeutic and clinical benefits.

### Bacterial vaccine candidates

The conserved nature and immunogenic properties of CDCs have made them very attractive candidates for vaccine development. This subsection reviews the application of major CDCs utilized as potential bacterial vaccine candidates, highlighting their specific inactivation method and their target (Table 1).

**Table 1:** Summary of major CDC-based bacterial vaccine candidates

CDCs	Bacterial source	Detoxification/modification method	Vaccine strategy	Reference
PLY	*Streptococcus pneumoniae*	Chemical (formaldehyde), Genetic modification.	Stand-alone antigen, fusion with PspA.	[22, 23]
LLO	*Listeria monocytogenes*	Genetic modification (undecapeptide mutations).	Stand-alone antigen.	[24]
SLO	*Streptococcus pyogenes*	Recombinant protein.	Multivalent vaccine (e.g., Combo4).	[25]
PFO	*Clostridium perfringens*	Recombinant protein, C-terminal domain.	Stand-alone antigen, combination vaccine.	[26]
SLY	*Streptococcus suiss*	Genetic modification (P353L mutation), recombinant protein.	Stand-alone antigen, adjuvant.	[27, 28]
PLO	*Trueperella pyogenes*	Genetic modification (W497F mutation).	Stand-alone antigen, preclinical model.	[29]

#### Pneumolysin (PLY) – *Streptococcus pneumoniae*

PLY is a major virulent factor that is secreted by *Streptococcus pneumoniae.* The toxin significantly contributes to the pathogenesis of the bacteria. PLY binds to cholesterol-rich membranes, resulting in the formation of transmembrane pores that lead to cell lysis and inflammation. The toxin is highly conserved across most pneumococcal strains, making it a prime target for vaccine development. However, its direct cytolytic effects mandates detoxification for safe use [30]. Previous studies over the last decade have identified chemical inactivation using formaldehyde and genetic modifications as detoxification methods to eliminate PLY hemolytic activity, making them safe candidates for pneumococcal vaccines development. A representative study developed a chemically detoxified PLY (dPLY) which retained its immunogenicity while eliminating toxicity [22]. In this study, immunization with dPLY in a mice model stimulated antibody protection capable of inhibiting PLY activity to provide protection against pneumococcal infection. Additionally, certain fusion proteins combining detoxified PLY with other pneumococcal antigens have been reported to enhance vaccine coverage. A recent study demonstrated that a detoxified pneumolysin derivative (PspA-PID) fusion protein induced cross-protective antibodies against multiple pneumococcal strains in mice [23].

#### Listeriolysin O (LLO) – *Listeria monocytogenes*

LLO is a CDC produced by *Listeria monocytogenes* that is released following the invasion of the host cell. LLO enables the bacterium to escape from the phagosome into the host cell cytoplasm [31]. LLO is highly immunogenic, and some derivatives have been investigated for their immunogenic properties. A detoxified LLO variant (LLO^T^) in which the CRM (critical for pore formation) was mutated has been shown to elicit a strong humoral response in mice [24]. However, the investigated LLO^T^ alone did not confer protection against *Listeria monocytogenes* infection. This indicates the need for combination with other potent antigens or adjuvants for effective vaccination.

#### Streptolysin O (SLO) – *Streptococcus pyogenes*

SLO is produced by *Streptococcus pyogenes.* The toxin significantly contributes to the pathogenesis of the bacteria as a strong virulent factor for host cell lysis [32]. SLO is unique and conserved for most *Streptococcus pyogenes* strains. Several vaccine candidates incorporating detoxified SLO are in preclinical development. A classic example is the Combo4 candidate currently being developed by GlaxoSmithKline PLC [25]. The Combo4 vaccine candidates contains recombinant SLO along with other antigens, which is currently being developed for broad coverage against *Streptococcus pyogenes* strains.

#### Perfringolysin O (PFO) – *Clostridium perfringens*

PFO have been implicated in the pathogenicity of *Clostridium perfringens*, causing diseases such as gas gangrene and enterotoxemia [33]. This toxin disrupts cell integrity by forming pores in cholesterol-rich membranes. A recent study demonstrated the disruptive effect of PFO on gastrointestinal epithelial cells [34]. Due to their ability to trigger release of antibodies during active infection, PFO have become useful in vaccine research as an immunogen. Recombinant PFO (WTrPFO) and its non-toxic C-terminal domain (rPFOC-ter) were found to be capable of eliciting a robust immune response in mice following immunization [26]. This study served to indicate the potential of PFO incorporation in vaccines against *Clostridium perfringens* infections.

#### Suilysin (SLY) – *Streptococcus suis*

SLY is secreted by the swine pathogen *Streptococcus suis*. SLY damages host cell tissues through formation of stable pores in cholesterol-rich membranes during active infection [35]. *Streptococcus suis* is an emerging zoonotic pathogen that causes diseases such as septicemia and meningitis, with SLY playing a major role in these infections [36]. The strong immunogenic properties of SLY have facilitated their emerging application in vaccine development. A genetically modified non-hemolytic mutant of SLY (rSLY(P353L) which retains immunogenicity while reducing pro-inflammatory responses has been reported since 2013 [27]. A recombinant form of suilysin (rSly) has been investigated recently as a potential vaccine candidate against *Streptococcus suis* [28]. In this study, immunized pigs showed appreciable IgG responses as well as elevated interferon-gamma (IFN-γ), and increased CD4⁺/CD8⁺ T cells which resulted in significant protection.

#### Pyolysin (PLO) - *Trueperella pyogenes*

PLO is secreted by *Trueperella pyogenes.* The toxin triggers serious immune responses beyond their cytolytic activity as CDCs [37]. A non-hemolytic mutant of pyolysin (PLOW497F), induced a significant immune response and provided full protection against *Trueperella pyogenes* infection in mice [29]. This finding supports PLO’s potential as a promising bacterial vaccine.

The above CDCs have been significantly explored as emerging candidates for vaccine development. Others such as anthrolysin O (ALO) [38] and intermedilysin (ILY) [39] secreted by *Bacillus anthracis* and *Streptococcus intermedius* respectively, remains relatively underexplored despite their known immunogenicity and well-defined role in bacteria pathogenesis. Ongoing and future studies may significantly enhance the exploitation of CDCs in vaccine development.

### Ligands for therapeutic applications

In recent years, CDCs have gained progressive attention as useful ligands for therapeutic application, primarily due to their unique ability to enhance endosomal and lysosomal escape. This unique property of CDCs is particularly significant for facilitating the intracellular delivery of potent therapeutics such as nucleic acids and chemotherapeutic agents. Among these CDCs, previous research has exhibited significant attention for LLO. The majority of these studies aimed to exploit the pH-dependent pore-forming activity of the toxin, which is activated in acidic environments of endosomes, promoting the release of encapsulated therapeutic agent into the cytosol [16].

There has also been notable research into LLO conjugation with various nanocarriers including liposomes and gold nanoparticles to enhance the delivery of therapeutic agents. The co-encapsulation of LLO with doxorubicin in liposomes has been demonstrated to enhance cytotoxicity against tumor cells by facilitating the drug’s escape from endosomes [40]. Similarly, another study combined gold glyconanoparticles with LLO peptide, which elicited boosted immune responses and effectively treated experimental bladder tumors [41]. Despite the facilitation of endosomal escape, recent studies have also shown and exploited the direct antitumor properties of CDCs. LLO has been utilized for the development of potent immunotoxins that selectively targets and kill tumor cells in acidic environment, thus reducing damage to healthy tissues [42]. CDCs also act as adjuvants in cancer immunotherapy, promoting valuable immune responses against tumor antigens [43].

Other CDCs such as SLO and PLY have been reported for potential utilization as ligands for therapeutic purposes, although to a lesser extent than LLO. A 2020 review highlights the use of SLO as transient permeabilizers to deliver macromolecules into cells, supporting their potential for therapeutic delivery [44]. PLY has also been reported in assisting in membrane permeabilization by disrupting cellular and endosomal membranes [45]. This mechanism can potentially enhance cytosolic delivery in nanoparticle systems.

However, the application of CDCs in therapeutics is not without drawbacks. Their cytotoxic nature poses risks to healthy tissues, and their incorporation into nanoparticles may compromise the structural integrity of the delivery system. In attempt to mitigate this challenge, current research has been focusing on developing potent non-cytotoxic mutants of CDCs that retain their functional properties.

### Cell membrane biosensors and biomarkers for bacterial sensing

CDCs have emerged in recent times as valuable tools for probing the distribution of membrane cholesterol. Their unique affinity and selectivity for cholesterol-rich domains has facilitated the visualization and quantification of cholesterol in various cellular systems. LLO has been studied and reported for use in identifying cholesterol-rich microdomains, which has aided in the study of the structure and dynamics of lipid rafts and membrane organization [46]. As previously stated, there is an associated cytotoxicity with the use of CDCs. To facilitate their use for this purpose, CDCs can be modified by altering their structural requirement which can influence their lytic activity. This could make CDCs more useful as biosensing probes without compromising the viability of cells. There are different detection methods that have been employed for CDC-based biosensors as depicted in Table 2.

**Table 2:** Recent applications of CDCs in biosensing

CDCs	Modification	Detection method	Application	Reference
LLO	Native LLO	Stimulated Emission Depletion Fluorescence Correlation Spectroscopy (STED-FCS).	Detection of lipid domain reorganization.	[47]
LLO	Recombinant LLO (rLLO), synthetic LLO peptides (LLO-1 and LLO-2)	ELISA-based assay.	Detection of listeriosis.	[17]
LLO	Native LLO	Colorimetric assay utilizing liposome-gold nanoparticle platform.	Rapid LLO detection. Suitable for point of care test.	[48]
PLY	PLY gene	Electrochemical detection using phenol red.	Sensitive and rapid detection of *Streptococcus pneumoniae*. Potential for point of care diagnosis.	[49]
PLY	Native PLY	Lateral Flow Immunoassay (LFIA) with magnetic nanoclusters.	Sensitive and rapid method for detecting *Streptococcus pneumoniae*.	[50]
SLO	Use of native antigens including SLO	Triplex immunoassay.	Efficient and streamlined method for detecting Group A *streptococcus* antigens including SLO.	[51]
SLO	Native SLO	Liposome-based fluorometric and colorimetric method.	Sensitive and portable method for detecting SLO toxins.	[52]
VLY	Native VLY	ELISA-based diagnostic test.	Accurate and easy diagnosis of bacterial vaginosis.	[53]
PFO	Native PFO	Liposome-based second harmonic scattering.	Non-invasive study of protein-membrane interactions.	[54]

Beyond cell membrane cholesterol probing, CDCs have also been exploited for detecting CDC-expressing bacterial infections. CDCs such as PLY, LLO, vaginolysin (VLY), SLO, have been detected using conventional methods such as enzyme-linked immunosorbent assays (ELISA), electrochemical methods, and immunoassays. Other sophisticated methods involving the use of nanoparticle-based approaches such as magnetic nanoclusters and liposome-gold nanoparticle platforms have also been exploited.

However, notable challenges are associated with the use of liposome-based platforms. Although liposomes are biocompatible, they often offer poor stability and rapid clearance. Recent studies have suggested that hybrid systems such as liposome-gold composite platforms or magnetic-liposome conjugates can mitigate these challenges. Conjugating liposomes with magnetic nanoparticles may enhance stability and sensitivity detection [55].

CDCs are now becoming integral to the design of biosensors and biomarker detection systems for potential bacterial sensing. Their application in this regard spans through cholesterol detection, lipid raft analysis, and diagnosis of bacterial infections. Their non-lytic interactions via modification, as well as their versatility in conjugation with nanoparticles make these toxins potentially suitable for real-time biosensing of bacterial infections.

### Targets for inhibition

CDCs have become useful targets for therapeutic inhibition due to their unique role in bacterial virulence and pathogenesis. This could either be a direct or indirect inhibition strategy. An indirect approach which may involve blocking host cell receptors or modifying membrane cholesterol content can reduce CDC activity. However, direct strategies have been majorly exploited for therapeutic inhibition of CDCs. This approach involves the use of monoclonal antibodies, small molecule inhibitors, or sequestration of the bacterial toxin.

#### Monoclonal antibody-based approaches

Monoclonal antibodies have recently emerged as a promising approach for CDC inhibition and neutralization. They bind to specific regions of the toxins, thereby preventing their interaction with the cholesterol-rich membranes of host cells. The cytosolic function of PLY has been inhibited using monoclonal antibodies that bind to L1 and L3 within D4, critical for cholesterol binding [56]. Monoclonal antibodies have also been utilized to inhibit the cytosolic functions of VLY produced by *Gardnerella vaginalis*, by binding to specific regions essential for the toxin’s activity [57]. This offers a potential therapeutic alternative for bacterial vaginosis. While the use of monoclonal antibodies offers high specificity and potency, notable drawbacks include high production cost, limited tissue permeability, poor stability via oral administration, and potential immunogenicity [58, 59]. These limitations have necessitated the exploration of alternative approaches including the use of small molecule inhibitors.

#### Small molecule inhibitors

The use of novel small molecules has been extensively exploited for inhibition of CDCs (Table 3). They represent attractive alternatives for monoclonal antibodies due to their relatively low cost of production [60]. Their minute nature also provides better tissue penetration and bioavailability. Recent studies have identified several small molecules that inhibits CDCs.

**Table 3:** Selected small molecule inhibitors targeting CDCs

CDC type	Inhibitors	Source	Target	Mechanism of inhibition	Reference
PLY	Shionone, Dryocrassin ABBA, Matcha green tea extract, Hederagenin, Acacetin, Wogonin, Quercetin, Hydrolysable tannins, Betulin.	Natural origin: triterpenoids, flavonoids, polyphenols, and phloroglucinol derivatives.	D1, D2, D3, D4, monomeric and oligomeric units.	Bind PLY subunits and domains to block pore formation. Inhibit hemolytic activity, reduce epithelial damage and inflammation. Direct antibacterial or anti-apoptotic activity.	[61–69]
LLO	Cinnamon Twig Extract, Atractylodin, Amentoflavone, Acacetin, Morin, Betulin, Kaempferol, Bioactive eucalyptus fractions.	Natural plant-derived flavonoids, triterpenoids, and extracts.	D1, D3, D4, monomeric and oligomeric LLO.	Direct binding to LLO subunits. Inhibit pore formation and hemolytic activity, reduce inflammation, oxidative stress, apoptosis, and bacterial virulence (in vitro and in vivo).	[70–77]
SLY	Quercetin, Formononetin, Acacetin.	Natural flavonoids and isoflavones.	SLY monomers, D2-D4 interface.	Inhibits SLY’s pore formation and hemolytic activity, reduces cytotoxicity and inflammation.	[78–81]
PFO	Verbascoside	Natural (phenylpropanoid glycoside)	Targets and inhibits PFO monomer	Inhibits PFO’s hemolysis and pore formation. Reduces tissue damage and mortality in mouse models. Anti-inflammatory and antioxidant properties.	[82]
SLO	Human Serum Albumin (HSA)	Natural (Human-derived plasma protein)	D2	Binds directly to SLO. Inhibits SLO pore formation. Decreases SLO- mediated cytotoxicity and hemolysis in vitro.	[83]

Some of the listed small molecule inhibitors can inhibit multiple CDCs. Acacetin inhibits both PLY, LLO, and SLY [65, 73, 81], while Quercetin inhibits both PLY and SLY [67, 78]. Betulin also inhibits both PLY and LLO. Amentoflavone is a flavonoid compound which inhibits LLO’s pore formation and hemolysis [72]. In addition, amentoflavone has been reported to inhibit PLY-induced cytotoxicity and hemolytic activity by specifically targeting the D3 and D4 domains [84]. Morin has an inhibitory effect against LLO oligomers [74]. Morin may also neutralize SLY-induced pore formation and hemolysis via D2 antagonism, preventing the transformation of SLY monomers to oligomer forms [85]. Previous studies have also reported that naturally produced molecules such as ApoB-100 lipoprotein, peptidoglycan, and the levofloxacin-ceftriaxone antibiotic combination therapy inhibit PLY-mediated pore formation, toxin release, and cytolytic as well as inflammatory activities [86–88].

#### Toxin sequestration strategies

Toxin sequestration involves the strategic capturing of CDCs before their interactions with host cell membrane. This strategy helps to neutralize the pathogenic effects of CDCs by preventing high levels of toxin release into the extracellular environment. This method employs the use of nanomaterials which mimic the host cell membrane and serve as decoy targets for the toxin.

##### Thin films and nanodroplets

Lipid nanodroplets and polymeric thin films have recently emerged as potential platforms for mitigating CDC-induced cell damage. They have been explored and utilized for their ability to adsorb, trap, and neutralize CDCs. These systems mimics and exploit the affinity of CDCs for lipid-rich host cell membranes, thereby enabling their localized neutralization of toxins before pore formation occurs in host cell membranes. Triglyceride-based lipid nanodroplets have been demonstrated to be kinetically stable and capable of forming a lipid-rich interface that can interact with specific proteins, thereby reducing their availability to interact with host cell membranes [89]. This platform enhances toxin capturing, while remaining physically stable in biological systems. Similarly, polymeric thin films mimicking cell membranes have shown effective adsorption of CDCs such as PLY, with plasma proteins, which modulates bacterial adherence and surface interactions of cells [90]. This platform provides adjustable physicochemical properties such as surface charges, lipid composition, and hydrophobicity, which can be optimized to selectively bind CDCs, while maintaining biocompatibility. Hybrid lipid bilayer films deposited on metallic surfaces have been developed, which further exploits this concept by combining mechanical stability of platform with membrane-like functions, thereby offering localized protection at platform interfaces [91].

##### Nanosponges

Nanosponges are biomimetic nanoparticles that are enveloped with red blood cells. They function as decoys for pore-forming toxins, including CDCs. They can efficiently absorb and neutralize CDCs. The use of nanosponges offers a widespread approach to toxin sequestration and neutralization. Erythrocyte-derived nanosponge have been explored for the sequestration and neutralization of pore forming toxins such as PLY secreted by *Streptococcus pneumoniae*, thereby preventing toxin-mediated membrane disruption [92]. This approach enables a wide range of toxin sequestration without directly targeting bacterial viability, which presents nanosponges as versatile platform for CDC neutralization.

Compared to other inhibition strategies, CDC sequestration offers the advantage of a reduced risk of resistance development, as it does not exert direct pressure on the bacteria. In addition, these nanomaterials can be used in conjunction with antibiotics to enhance therapeutic efficacy without inducing cell lysis, which could lead to the release of lethal levels of toxin.

### Therapeutic release

CDCs primarily target and disrupt host cell membranes by forming stable transmembrane pores. This unique feature of CDCs has been exploited in the design of emerging therapeutic delivery systems that leverage their pore-forming ability on cholesterol-rich membranes. While CDCs are traditionally viewed as targets for neutralization, their ability to create stable pores has also been harnessed in therapeutic delivery. Recent advancements in nanotechnology have facilitated the development of responsive delivery systems that exploit CDC activity for stimuli-responsive drug release. This has enhanced targeted therapeutic strategies and selective toxicity.

**Fig. 3:**
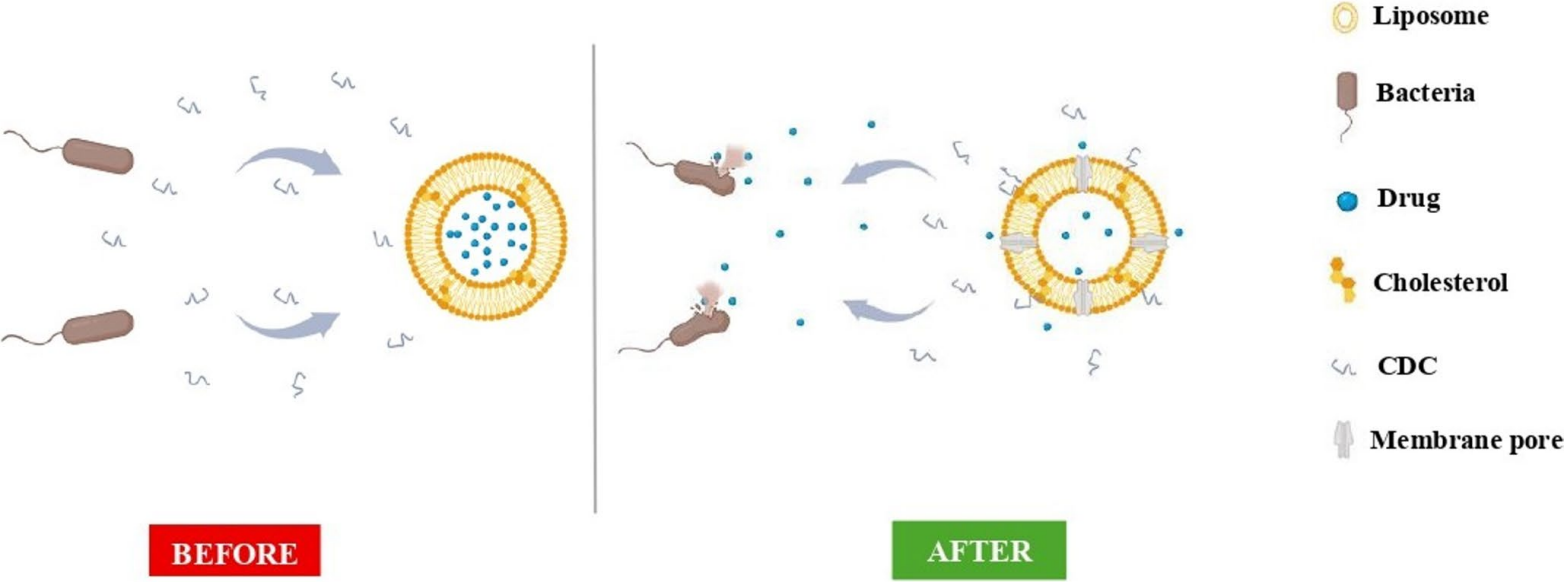
Schematic representation of CDC-responsive therapeutic delivery. Illustrating how pore- formation by CDCs triggers the release of encapsulated liposomal payload

Liposomal formulations represent the most studied nanomaterials for CDC sequestration and therapeutic toxin neutralization. Engineered liposomes enriched with cholesterol and phospholipids have been designed to act as nanotraps that sequester CDCs, and effectively neutralizing their pore forming activity and protecting host tissues from damage caused by toxin [93]. Liposome-based responsive systems have been utilized for the targeted delivery of antimicrobial agents. Liposomes are versatile nanocarriers, which are known for their biocompatibility and ability to encapsulate both hydrophilic and hydrophobic drugs. Some studies have demonstrated that the pore-forming activity of CDCs can be exploited to trigger the release of liposomal payloads via pore formation on cholesterol-rich liposomal vesicles (Fig. 3). This approach triggers the release of liposomal contents in an infected environment. Lemongrass essential oil encapsulated in cholesterol-rich liposomal vesicles has been reported to exhibit sustained release and antibacterial activity against *Listeria monocytogenes* in food models [94]. More recently, a study reported the exploitation of a PLY-responsive cholesterol-rich liposomal system for the selective treatment of *Streptococcus pneumoniae* infection [21]. Similarly, bacteria-responsive liposomes have been developed to release their cargo in response to VLY produced by *Gardnerella vaginalis* [20]. These liposomes remain stable under normal conditions but release their contents upon toxin detection, which demonstrates a targeted and controlled approach for therapeutic delivery.

Calcium phosphate (CaP) nanoparticles used for toxin-triggered delivery, possess desirable properties such as biodegradability and pH sensitivity, and have been widely studied for their intracellular delivery. Their potential to dissolve in acidic endosomal environments makes them unique and ideal for cytosolic delivery of biomolecules. They can be engineered with peptides that target CDCs and facilitate toxin-induced intracellular activity. CDC-targeting CaP nanoparticles as nanocarriers for intranasal delivery of peptides have been reported for the potential treatment of pneumococcal infection in zebrafish and mice models [95].

These responsive systems demonstrate the feasibility of exploiting the CDC-producing bacterial virulence mechanisms for advanced targeted drug delivery. This approach offers advantages of precise, targeted, localized, and sustained delivery, while minimizing systemic side effects and toxicity.

## Factors for development and evaluation of CDC-responsive systems

The development of CDC-responsive systems requires consideration of several factors to ensure effective targeting, release, and clinical potential. These factors are carefully considered regardless of the platforms or systems employed. A critical factor in the design of CDC-responsive system is the determination of specific recognition or binding to CDC toxins. It is well established that CDCs exert their effect by binding to cholesterol-rich domains and oligomerizing to form stable pores. Effective CDC-responsive systems are designed to target the stages of CDC-membrane interaction. Understanding the molecular structure of CDCs and their interactions are thus essential for designing effective CDC-responsive systems and delivery platforms that specifically recognize and bind CDC toxins.

Stimuli-responsive release represents another factor. Stimuli-responsive carriers encapsulate active pharmaceutical ingredients (APIs) and undergo structural modifications to release their payloads in response to specific triggers [96]. Encapsulation strategies such as the use of liposomes and CaP nanoparticles which are engineered to respond to CDC-triggered membrane disruption have demonstrated effective therapeutic release in proximity of infection sites. These systems leverage the toxin requirement for cholesterol to interact with host cell membranes.

Biocompatibility also represents another important consideration in the development of CDC-responsive systems. The biocompatibility and safety evaluation of developed platforms are significant and must be considered in the design of responsive delivery systems [97]. Efforts are focused on the use of naturally derived FDA-approved materials like phospholipids or other biodegradable polymers to ensure in vivo safety of the designed systems. The materials utilized must be non-immunogenic and biodegradable into safe byproducts. CDC-responsive systems are not only considered for their efficacy but also biosafety.

Bearing in mind the mechanism of CDC interaction with host cell membrane and their role in the pathogenicity and virulence of CDC-expressing bacteria, the evaluation of developed CDC-responsive systems will typically include assessing toxin recognition and binding, release kinetics of the developed platform, biodistribution, therapeutic efficacy, and immunogenicity assessment. Preclinical studies considering these evaluation criteria are very critical in the development and advancement of CDC-responsive system for biomedical application.

### Toxin isolation and detection

CDCs are isolated in a pure and active form to study their properties and potential for therapeutic applications. Centrifugation and various chromatographic techniques have evolved and now represent standard biochemical methods for the purification of toxin proteins [98, 99]. The toxins are isolated and highly purified to ensure accuracy of biological assays. Previous studies have also shown that activation of highly purified toxin is achieved by the addition of reducing agents like dithiothreitol (DTT), which restores the disulfide bonds and facilitates toxin activity [100, 101].

Hemolysis assays remain the gold standard for the in vitro detection of CDCs, where the degree of erythrocyte lysis with incubated toxins are evaluated. Hemolysis assays have been used to assess and confirm the pore-forming activity of discoidinolysin, a CDC produced by *Streptococcus mitis* [102]. As previously discussed, detection and quantification of toxin could also be achieved via ELISA-based techniques. Advanced technologies such as electrochemical sensors have been developed for rapid toxin detection in complex biological systems [103]. This may find promising potential for detection of CDCs.

The in vivo detection of CDCs often employs the use of advanced imaging techniques to determine the presence of the toxin within tissues and systemic circulation. This typically involves approaches that leverage the affinity of CDCs for cholesterol-rich domains. A recent innovative method exploited a split-fluorescent protein sensor derived from PFO to visualize membrane cholesterol in mice models [104]. The sensor’s interaction with cholesterol facilitated the detection of CDC activity within tissues. This method represents a promising approach for studying the in vivo interactions of CDCs. Other analytical techniques such as mass spectrometry-based and antibody-based approaches, may also be exploited for the in vivo detection of CDCs. This technique does not only confirm infection but may also evaluate the severity of exposure based on toxin load.

CDCs are inactivated in a very cautious manner to retain their immunogenic properties, while eliminating cytotoxicity. Heat treatment has been previously reported as an effective method for significantly reducing the hemolytic activity of CDCs such as SLY, PLY and SLO [105]. As indicated earlier, chemical inactivation has also been utilized, although genetic detoxification via mutation has been more widely exploited due to its favorable outcomes. Overall, effective purification, isolation, detection, and inactivation techniques are essential for the study and biomedical application of CDCs.

### Nanomaterials for facilitation of CDC interactions

The use of nanomaterials represents an emerging and promising strategy in mediating and facilitating interactions with CDCs. Various nanomaterials have been utilized for CDC interactions (Table 4). The use of these materials enhances therapeutic efficacy through mechanisms such as toxin sequestration and neutralization via a controlled release approach. The design of these platforms with consideration of their composition, particle size, surface charge and functions, greatly influences their interactions with CDCs as well as their ability to penetrate microbial biofilms.

**Table 4:** Overview of nanomaterials for CDC interaction

Nanomaterial type	Examples	Key features	Mechanism of CDC interactions
Inorganic	CaCO₃, Metallic (gold and silver).	Biocompatibility, pH-responsive dissolution.	CDC sequestration and membrane disruption.
Polymeric	PLGA, PLGA-PEG.	Biodegradability and controlled release.	Encapsulation and targeted delivery of CDC inhibitors.
Organic	Liposomes, membrane vesicles.	Mimics host cell membrane, appropriate cholesterol content.	CDC sequestration and neutralization.
Hybrid	Lipid-coated, HLs.	Improved structural integrity and multifunctionality.	Enhanced targeted delivery and biofilm penetration.

#### Inorganic nanomaterials

Inorganic nanomaterials including calcium carbonate (CaCO₃) and metallic nanoparticles have been exploited for their potential to deliver APIs specifically within CDC-active microenvironments. The biocompatibility and pH-responsive dissolution of CaCO₃ nanoparticles can be beneficial for targeted delivery in acidic infection sites [106]. Metallic nanoparticles such as those composed of gold and silver, possess inherent antimicrobial properties which can also facilitate bacterial membrane disruption [107, 108]. This approach represents an emerging strategy and further enhances the neutralization of CDCs.

#### Polymeric nanomaterials

Polymeric nanomaterials, typified by those containing poly(lactic-co-glycolic acid) (PLGA), have been found capable of facilitating effective interactions with microbial membranes and biofilms [109]. These nanomaterials are biodegradable and can be designed to encapsulate CDC inhibitors and other APIs, thereby facilitating controlled and targeted delivery. In addition, surface modification of these materials such as the incorporation of polyethylene glycol (PEG), may enhance circulation time and reduce immunogenicity [110, 111]. Hybrid systems containing PLGA and lipid have demonstrated enhanced stability and bioavailability [112], eliciting improvements to both biofilm penetration and CDC neutralization.

#### Organic nanomaterials

Organic nanomaterials including liposomes and membrane vesicles have been extensively explored as promising platforms to facilitate interactions with CDCs. They are very effective in their interactions with CDCs due to their lipid composition. As previously stated, liposomes can mimic host cell cholesterol-rich membranes to sequester released CDCs. In this platform, the incorporation of cholesterol is crucial for interaction with CDCs, due to their inherent dependence on membrane sterol content. Increased cholesterol content in liposomal vesicles enhances their binding and interactions with CDCs, leading to more effective CDC neutralization.

#### Hybrid nanomaterials

Hybrid nanomaterials which combine both inorganic and organic nanoparticles may offer multifunctional systems for improved CDC interaction. Lipid-coated hybrid nanoparticles have been reported to offer enhanced structural integrity through their inorganic cores, while the lipid vesicles contribute to improved biocompatibility and broad functional versatility [113]. Hybrid liposomes (HLs) have also been reported for their potential for improved stability, multifunctionality, and therapeutic versatility including theragnostic applications [114]. This system also represents promising potential for the design of platforms to enhance biofilm penetration and controlled delivery of APIs targeting CDCs.

The physicochemical properties of nanomaterials, including particle size and surface charge, play crucial roles in their interactions with CDCs and microbial biofilms. Nanomaterials with small particle sizes have been reported to interact effectively with bacterial membranes and exhibit superior biofilm penetration. Nanocarriers with particle size smaller than 130 nm can effectively penetrate biofilm matrices [115]. The surface charge also influences interaction. Positively charged nanoparticles have been shown to effectively bind and interact with negatively charged bacterial membranes, and this enhances the therapeutic efficacy of the platform [116].

The cholesterol content of the designed platform represents another important factor mediating CDC binding and interaction. Specifically, it has been previously reported that liposomal membranes require a cholesterol concentration between 50 and 55 mol% for maximal binding and activity [117]. Increasing the cholesterol content in lipid membranes also facilitates CDC binding and pore-forming activity [118]. Designing nanomaterials with appropriate cholesterol content is critical and necessary for effective CDC sequestration and neutralization.

The design of nanomaterials with careful consideration of composition, particle size, surface charge, cholesterol content, and functions are indispensable for effective interactions with CDCs. Recent advancements in use of hybrid systems with strategic surface modifications continue to enhance the therapeutic potential and application of these systems in toxin neuralization and prevention of associated bacterial infections.

### Bacterial targeting and treatment

The growth state of CDC-expressing bacteria and their surrounding microenvironmental conditions such as pH and oxygen level, largely influences the expression and activity of CDCs. Notably, VLY produced by *Gardnerella vaginalis* have been reported to be expressed differently in planktonic cultures and biofilms. Elevated expression of VLY in planktonic state compared to biofilm-associated bacteria has been reported [119]. This may be a unique strategy for bacteria to bypass host immune detection to facilitate chronic colonization. Studies have also reported that CDCs exhibit pH-dependent activity, as well as oxygen levels modulating CDC expression. The pore-forming activity of inerolysin (INY, produced by *Lactobacillus iners*) and LLO is enhanced in acidic pH levels [120], while hypoxic conditions considerably reduce PLY’s pore-forming capacity with cholesterol-rich membranes [121].

While in vitro studies have been beneficial in studying CDCs, they do not replicate the ideal complexity of in vivo conditions. Factors such as variations in CDC-expressing bacterial species, host immune interactions, as well as varying pH levels in various tissues can significantly influence the in vivo expression and activity of CDCs. This may also affect the targeted treatment of CDC-expressing bacterial infections. Emerging nanotechnology-based approaches for mitigating these challenges have been explored for targeted delivery and effective treatment [122]. Nanoparticles can be designed with specific modifications of their physicochemical properties to directly facilitate their interactions with bacterial membranes and biofilms.

The route of administration of these nanoparticles also plays an important role in achieving successful cell-targeted specificity at infection sites [123]. The specific route of administration for enhanced potential of therapeutic efficacy may be dependent on the specific site of infection [124, 125]. Common routes of administration include pulmonary, oral, transdermal, and intravenous delivery. For respiratory infections caused by *Streptococcus pneumoniae*, the pulmonary route via inhalation may be utilized for localized and targeted delivery to the lungs, whereas most gastro-intestinal and systemic infections primarily benefit from oral and intravenous administration.

Despite the emerging and promising developments in the use of nanomaterials for targeted delivery and treatment of CDC-producing bacterial infections, there is a significant need for comprehensive in vivo studies to better understand the activity of CDC-mediating infections across different microbial communities and under varied physiological or environmental conditions. This will positively impact on the design of more effective nanomaterials for treatment of CDC-associated diseases.

### Characterization of toxin interactions

**Fig. 4:**
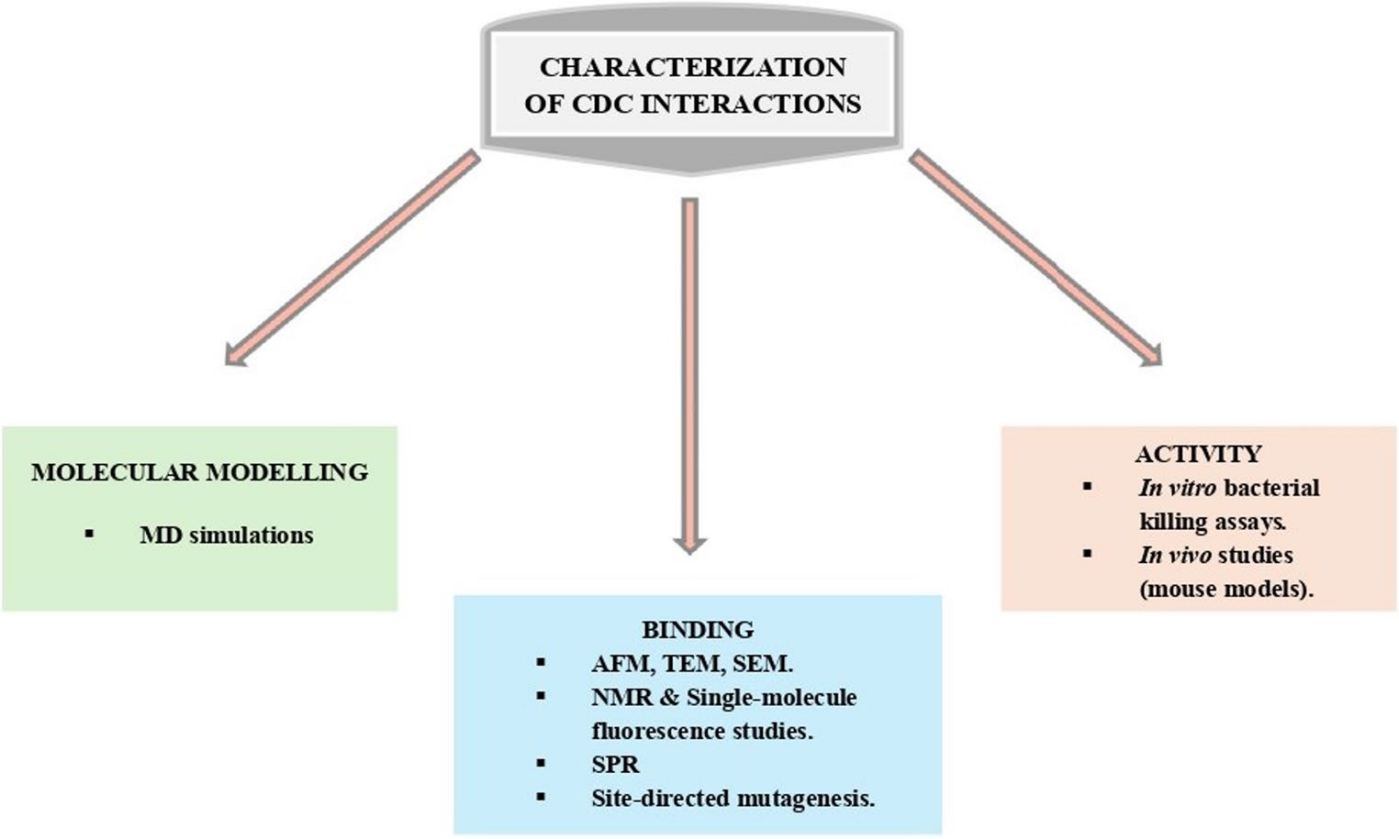
Summary of characterization of CDC interactions

The development of effective therapeutic and diagnostic strategies for CDC-associated infections requires a comprehensive understanding of CDC interactions at the molecular level, including their binding mechanisms and functional activities. Characterizing the interactions between CDCs and nanomaterials is essential for the development of effective therapeutic and diagnostic strategies. Characterizing CDC interactions involves investigating their molecular dynamics, binding mechanisms, and functional activities (Fig. 4).

#### Molecular modelling

Recent advancements in molecular dynamics (MD) simulations have provided valuable insights into the role of cholesterol in modulating membrane properties and have facilitated the investigation of protein-lipid interactions in relation to disease mechanisms [126]. The recent exploration of MD simulations has been instrumental in the unraveling and elucidation of interactions between CDCs and lipid membranes. MD simulation has been utilized to explore how LLO interacts with lipid bilayers composed of 1,2-dioleoyl-sn-glycero-3-phosphocholine (DOPC) and cholesterol [127]. This simulation revealed how LLO induces lipid reorganization and cholesterol within the membrane, which provides detailed molecular insight into the mechanism of membrane disruption and pore formation by CDCs. This insight is very critical for the design of nanomaterials that can effectively interact and neutralize CDCs.

#### Binding

Various techniques have been explored to study the binding of CDCs to membrane cholesterol and morphological characteristics of associated nanomaterials. While atomic force microscopy (AFM), transmission electron microscopy (TEM), and scanning electron microscopy (SEM), provides morphological elucidation of designed nanomaterials [128, 129], the use of nuclear magnetic resonance (NMR) and single-molecule fluorescence studies may offer detailed information on binding dynamics and associated conformational changes [130, 131]. Surface plasmon resonance (SPR) have also been used to measure real-time binding kinetics and affinities between CDCs and cholesterol-rich vesicles [132]. Identification of key amino acid residues involved in cholesterol binding and CDC activity via site-directed mutagenesis has also been reported [133]. These techniques collectively contribute to understanding CDC binding mechanisms and interactions.

#### Activity

Investigating the functional activities of CDCs can be achieved via both in vitro and in vivo studies. In vitro bacterial killing assays have been utilized to investigate the release kinetics and efficacy of CDC-targeting agents and designed nanoplatforms [134]. As highlighted previously, in vitro studies such as mouse models have been employed to assess the activities and therapeutic potential of CDC-targeting strategies, as well as the potential development of CDCs as bacterial vaccine candidates. Overall, a holistic approach encompassing molecular modelling, binding studies, and activity assays is essential for the comprehensive characterization of CDC interactions. This approach will provide valuable insight into the design and development of effective CDC-targeting nanotherapeutics.

## Challenges in clinical translation

CDCs remain pivotal virulence factors produced by Gram-positive bacteria which have significantly contributed to challenging diseases such as pneumonia, soft tissue infections, and bacterial meningitis. Despite extensive in vitro and animal model studies elucidating CDC binding mechanisms and interactions, the biomedical application and clinical translation of CDC-targeting nanomaterials remain limited. Several challenges impede the clinical translation of CDCs. While numerous in vitro studies of CDCs exist, there remains limited in vivo data and clinical trials assessing CDC-associated therapeutics. This research gap hinders the comprehensive understanding of the in vivo interactions of CDCs, their therapeutic potential and safety profiles in complex biological systems and human microenvironments. CDCs are also associated with biofilm-producing bacteria, which present a major challenge for therapeutic interventions. Biofilms are known to hinder the penetration of potential therapeutics. The biofilm microenvironments may suppress CDC expression, thereby reducing the therapeutic efficacy of CDC-targeting platforms. However, dual-species biofilms have been reported to upregulate CDC expression and activity [135]. The specific concentration of CDCs during active infections may also vary depending on pathogen type, site of infection, and particular stage of disease progression. Recent studies on CDC-associated infections and therapeutics may utilize concentrations that may not actually reflect those of real-time clinical situations, potentially leading to underestimation or overestimation of therapeutic efficacy. The specific route of delivery can also present challenges in the distribution, efficacy, and safety of CDC-targeting nanotherapeutics. While respiratory infections caused by CDC-expressing bacteria may benefit from pulmonary delivery, oral and intravenous administration may be ideal for systemic infections. Each route has its own unique drawbacks including barriers to absorption, increased chances for off-target delivery, as well as patient compliance issues. Optimization of the physicochemical properties of CDC-targeting nanomaterials may also pose challenges for their potential clinical application. An ideal balance of nanomaterial sizes, surface charge, and hydrophilicity will significantly influence their interactions with bacterial membranes and biological systems, including their ability to effectively target and neutralize CDCs. Maintaining sustained release of CDC-nanotherapeutics at the specific site of infection, while significantly limiting potential resistance mechanism also present a challenge for the successful therapeutic utilization of CDC-targeting strategies. Additionally, the associated cost of developing and characterizing these strategies may affect their general accessibility and widespread adoption.

## Conclusions and future perspectives

CDCs offer significant prospects for the development of targeted therapeutic systems and biosensing platforms for bacterial infections. This has been demonstrated in various reported preclinical studies. The emerging application of CDCs for advanced targeted therapies including bacterial vaccine development, biosensors, ligands for therapeutic application, and its exploitation as potential target for inhibition, represents a promising development in biomedical innovation. Recent advancement in nanomaterial-based CDC-targeting platforms such as liposomes, polymeric nanoparticles, and hybrid systems, presents innovative strategies leveraging CDC interactions with biological systems to combat bacterial infections. However, the clinical translation of CDC-based therapies has been hindered by several factors. Key challenges such as bacterial biofilm complexity, variable toxin levels at infection site, limited applicability of preclinical studies, high cost of therapies, and the need to optimize the physicochemical properties of nanomaterials for improved penetration, targeting, and sustained efficacy, pose significant challenges to clinical translation. Attempts towards resolving these barriers are essential for clinical application and broad utilization of CDC-based therapeutic systems.

Despite these challenges, there is significant potential for future progress. The development of more sophisticated and clinically relevant in vitro and in vivo studies that better reflect complex human microenvironments and infection conditions will considerably facilitate the evaluation and potential biomedical application of CDC-targeting systems. Emerging developments in nanotechnology, particularly the use of responsive and biocompatible nanoplatforms are expected to play a critical role in enhancing the effectiveness of CDC-based therapeutics and supporting their large-scale implementation. Ongoing research focusing on developing versatile and efficacious systems against various CDC-expressing bacteria, as well as improving the pharmacokinetics of these therapies, will positively impact their potential for successful clinical application.

The exploitation of CDCs for targeted biosensing and therapeutic systems is an emerging and promising area of research with valuable prospects for advancing the treatment of bacterial infections. While significant challenges remain, the combination of responsive nanomaterials for targeted delivery and an enhanced understanding of CDC mechanisms and interactions holds great potential for future milestones in this field.

## Data Availability

No datasets were generated or analysed during the current study.
